# Clinical significance of NLRP3 inflammasome and related cell molecules in early diabetic kidney disease in elderly population

**DOI:** 10.5937/jomb0-45950

**Published:** 2024-11-16

**Authors:** Xiaoli Li, Shiwei Liu, Jinrong Huangfu, Nannan Lai, Yan Shang

**Affiliations:** 1 Third Hospital of Shanxi Medical University, Shanxi Academy of Medical Sciences, Shanxi Bethune Hospital, Tongji Shanxi Hospital, Department of Endocrinology, Taiyuan, China; 2 Huazhong University of Science and Technology, Tongji Medical College, Tongji Hospital, Department of Endocrinology, Wuhan, China; 3 Third Hospital of Shanxi Medical University, Shanxi Academy of Medical Sciences, Shanxi Bethune Hospital, Tongji Shanxi Hospital, Department of Nephrology, Taiyuan, China; 4 Huazhong University of Science and Technology, Tongji Medical College, Tongji Hospital, Department of Nephrology, Wuhan, China

**Keywords:** NLRP3 inflammasome, early diabetes, kidney disease, clinical significance, NLRP3 inflamazom, rani dijabetes, bolest bubrega, klinički značaj

## Abstract

**Background:**

The paper aims to investigate the expression level of NLRP3 inflammasome and its related cell molecules in early diabetes kidney disease (EDKD) in the elderly and its clinical application value.

**Methods:**

From October 2021 to April 2023, 50 elderly patients with T2DM (T2DM group), 50 elderly patients with EDKD (EDKD group) and 50 elderly people who passed the health check-up (healthy group) were chosen as the study subjects. Plasma NLRP3 inflammasome and related cells (blood leukocyte count, monocyte count, lymphocyte count) molecular (NT-proBNP and others) levels are tested, and Pearson correlation analysis is utilized to explore the correlation among plasma NLRP3 inflammasome and related cells, molecules, and renal function indicators (UACR, BUN, Ucr) in elderly patients with EDKD.

**Results:**

(1) The three groups' comparison in HbA1c, FIns, HOMA-IR, UACR, BUN, Ucr, SOD, MCP-1, and TNF-a levels were with P<0.05. The levels of TG and LDL-C in the EDKD group were higher than those in the T2DM and the healthy groups; the levels of FPG, HbA1c, FINs, HOMA-IR, UACR, SOD, MCP-1, TNF-a in the EDKD and T2DM groups were higher than those in the healthy group, while SOD was smaller than that in the healthy group; the levels of BUN, Ucr, hs-CRP, FPG, HbA1c, FINs, HOMA-IR, UACR, SOD, MCP-1, TNF-a in the EDKD group were higher than those in the T2DM group, while SOD was smaller than that in the T2DM group. The above results were with P<0.05. (2) It has P<0.05 in Monocyte count, NLRP3, NT-proBNP, caspase-1, ASC and others in the three groups. Those in the EDKD and T2DM groups were higher than those in the healthy group. The levels of these indicators in the EDKD group were higher than those in the T2DM group, with P<0.05. NLRP3, Caspase-1, ASC, IL1b, and IL-18 were positively correlated with UACR, BUN, and Ucr in the EDKD group. All the above differences were P<0.05.

**Conclusions:**

NLRP3 inflammasome and its related molecules caspase-1, ASC, IL-1b, IL-18 and other levels increase in early elderly EDKD and are closely related to the severity of EDKD.

## Abstract (sr)

**Uvod:**

Rad ima za cilj da ispita nivo ekspresije NLRP3 inflamazoma i njemu srodnih ćelijskih molekula u ranoj fazi dijabetesne bubrežne bolesti (EDKD) kod starijih osoba i njegovu kliničku primenu.

**Metode:**

Od oktobra 2021. do aprila 2023. godine, izabrano je 50 starijih pacijenata sa T2DM (T2DM grupa), 50 starijih pacijenata sa EDKD (EDKD grupa) i 50 starijih osoba koje su prošle zdravstveni pregled (zdrava grupa). Testirani su nivoi inflamazoma NLRP3 u plazmi i srodni (broj leukocita u krvi, broj monocita, limfocita) molekularni (NT-proBNP i drugi) nivoi, a korišćena je Pirsonova korelaciona analiza za istraživanje korelacije između plazma NLRP3 inflamazoma i srodnih ćelija, molekula i indikatora renalne funkcije (UACR, BUN, Ucr) kod starijih pacijenata sa EDKD.

**Rezultati:**

(1) Poređenje tri grupe u nivoima HbA1c, FIns, HOMA-IR, UACR, BUN, Ucr, SOD, MCP-1 i TNF-a bilo je sa P<0,05. Nivoi TG i LDL-C u grupi sa EDKD su bili viši od onih u T2DM i zdravim grupama; nivoi FPG, HbA1c, FINs, HOMA-IR, UACR, SOD, MCP-1, TNF-a u grupama EDKD i T2DM su bili viši od onih u zdravoj grupi, dok je SOD bio manji od onog u zdravoj grupi; nivoi BUN, Ucr, hs-CRP, FPG, HbA1c, FINs, HOMA-IR, UACR, SOD, MCP-1, TNF-a u EDKD grupi su bili viši od onih u T2DM grupi, dok je SOD bio manji od nivoa u grupi T2DM. Gornji rezultati su bili sa P<0,05. (2) Ima P<0,05 u broju monocita, NLRP3, NT-proBNP, kaspazi-1, ASC i drugima u tri grupe. Oni u EDKD i T2DM grupama su bili veći od onih u zdravoj grupi. Nivoi ovih indikatora u grupi sa EDKD su bili viši od onih u grupi sa T2DM, sa P<0,05. NLRP3, kaspaza-1, ASC, IL-1b i IL-18 su bili u pozitivnoj korelaciji sa UACR, BUN i Ucr u EDKD grupi. Sve gore navedene razlike su P<0,05.

**Zaključak:**

NLRP3 inflamazom i njemu srodni molekuli kaspaza-1, ASC, IL-1b, IL-18 i drugi nivoi se povećavaju u ranoj dijabetesnoj bolesti bubrega kod starije populacije i usko su povezani sa ozbiljnošću EDKD.

## Introduction

Abnormal glucose metabolism in the body of diabetes can cause diabetes kidney disease (DKD). Clinical findings show that even if patients’ blood sugar and blood pressure are controlled, DKD cannot be avoided. Ineffective intervention will lead to its progress to end-stage kidney disease so that the disease is irreversible and threatens the patient’s life [1]. Therefore, early diagnosis and therapy of DKD is very important. Recent research reports indicate that the levels of IL-1β and IL-18 in the serum of patients with DKD are elevated [2]. The cleavage and activation process of those is dominated by the inflammasome of nucleotide-binding oligomerization domain-like receptors protein 3 (NLRP3), which includes NLRP3, apoptosis-related spot protein (ASC), and cysteine-containing aspartic acid protein hydrolase 1 (caspase- 1) [3] [4]. Peripheral monocyte ASC, caspase-1 and other molecules participate in the progressive activity process of diabetes [5]. There is little research on the changes of NLRP3 inflammasome and related cells and molecules in early diabetes kidney disease (EDKD) in the elderly. Thus, this study aims to analyze the expression level and clinical meaning of NLRP3 inflammasome and related cells and molecules in early EDKD and provide a basis for early differential diagnosis of EDKD.

## Materials and methods

### General materials

It selects 100 elderly patients with T2DM who visited our hospital from October 2021 to April 2023. Based on the ratio of urinary albumin to creatinine (ACR), patients with T2DM alone and without DKD (ACR<30 mg/g) are classified as T2DM group (n=50), and patients with EDKD (30 mg/g ACR<300 mg/g) are classified as EDKD group (n=50). Inclusion criteria: (1) according to the 2018 diabetes medical diagnosis and treatment standards of the American Diabetes Association [6], T2DM is diagnosed; (2) age 60 years old; (3) gentamicin, kanamycin, aciclovir, ibuprofen and other drugs with nephrotoxicity have not been used in the past year; (4) all patients are aware of this study and voluntarily participate in it. Exclusion criteria: (1) definite diagnosis of Type 1 diabetes; (2) acute complications of diabetes and chronic heart failure; (3) concomitant nephrotic syndrome, nephritis, acute and chronic infections, and immune dysfunction; (4) women who are breastfeeding or pregnant. Additionally, elderly individuals who passed the body check-up are chosen as the health group (n=50). T2DM, EDKD and Health groups: 28, 26, 23 males and 22, 24, 27 females, respectively, aged 63–78, 61–79 and 62–75 years, respectively, and with an average age of (67.25±5.16), (66.43±6.10) and (65.56±5.47) years, respectively. The comparison in gender and age is with *P*>0.05.

### Methods

All patients are taken 15 mL of elbow vein blood on the morning of admission, anticoagulated with heparin sodium, centrifuged and separated from the bleeding serum, and placed at -80°C for testing. The level of HbA1c is measured by a glycated hemoglobin analyzer and its matching reagents. An automatic biochemical analyzer measures FPG, BUN and Ucr levels, and a urine analyzer in the middle of clean urine measures the creatinine ratio (UACR). The TC, TG, and other levels are detected with Abbott Laboratories ARCHITECT C8000. Measurement of FINs levels uses a fully automated electrochemical luminescence analyzer. The special protein analyzer measures the hs-CRP level, and the hydroxylamine method (provided by Shanghai Huzheng Biotechnology Co., Ltd.) is used to measure the SOD level. ELISA method (all kits provided by Shanghai Enzymes Biotechnology Co., Ltd.) and supporting kits are used to determine FINs, NT-proBNP and others.

Monocyte is added to the EP tube without RNA enzyme, and then 1 mL of trizol is added. It needs to be shaken and placed on ice for 5 minutes. Then, precooled chloroform 200 μL is added. After severe vibration for 1 minute, 12000×g, it is centrifuged at 4°C for 20 minutes. The upper liquid is transferred, and 500 μL of isopropanol is added. After mixing, it is placed at 4 °C and centrifuged again. The lower layer substance (RNA) is taken, washed with 75% ethanol. 50 mL of deionized water (de RNA enzyme) is resuspended, and a reverse transcription system is prepared. The real-time quantitative PCR reaction is performed at 42°C for 60 minutes and 70°C for 5 minutes. Reaction system: SYBR Green 12.5 μL, both upstream and downstream primers are 0.5 μL, water 6.5 μL and complementary DNA 5 μL. PCR amplification environment: 95 for 10 minutes, 95°C for 15 seconds, 58°C for 1 minute, 72°C for 30 seconds, 40 cycles. Primer sequence reference literature [7].

### Statistical methods

SPSS 23.0 statistical software was utilized to process the data, and the measurement data was described by »x̄±s«. The comparison between the two groups was conducted using the t-test, and the comparison between the three groups was conducted using the F-test. The counting data is described in n (%) and subjected to χ^2^ test. Pearson correlation is applied to analyze the correlation between plasma NLRP3 inflammasome, IL-1β, IL-18 and UACR, BUN, and Ucr in elderly patients with early diabetes and renal disease. *P*<0.05 means that it has a statistically significant difference.

## Results

### Comparison of three sets of laboratory indicators

The levels of SBP, TG, LDL-C, HDL-C, hs-CRP, FPG, HbA1c, FIns, HOMA-IR, UACR, BUN, Ucr, SOD, MCP-1, and TNF-α in the three groups were compared, and the differences were statistically significant [P(TG)=0.018, P(LDL-C)=0.030, P(HDL-C)=0.001, *P*<0.001 for the rest]. TG, LDL-C, hs- CRP, FPG, HbA1c, FIns, HOMA-IR, UACR, SOD, MCP-1, and TNF-α in the EDKD group were higher than those in the healthy group [P(TG)=0.011, P(LDL-C)=0.020, *P*<0.001 for the rest] and higher than the T2DM group [P(TG)=0.036, P(LDL-C)= 0.031, P(TNF-α)=0.005, *P*<0.001 for all the rest]. hs-CRP, FPG, HbA1c, FIns, HOMA-IR, UACR, MCP-1, and TNF-α in the T2DM group were higher than those in the healthy group [P(UACR)=0.030, *P*<0.001 for the rest], and HDL-C and SOD were lower than those in the healthy group (P=0.011, *P*<0.001). BUN and Ucr in the EDKD group were higher than those in the T2DM group (*P*<0.001 for all) (see Table 1). This resultant data suggested that T2DM patients and EDKD patients have different degrees of abnormalities of blood glucose, insulin, and lipids, as well as hepatic and renal function damage, and this abnormal change was more obvious in EDKD patients.

**Table 1:** Comparison of three sets of baseline data (x̄±s). Note: TC=total cholesterol, hs-CRP=high-sensitivity C-reactive protein, FPG=Fasting blood sugar, HbA1c=glycated hemoglobin, FIns=fasting insulin, HOMA-IR=insulin resistance, SOD=superoxide dismutase, SBP=systolic pressure, MCP-1=monocyte chemotactic protein, DBP=diastolic pressure, TG=triglyceride, BUN=blood urea nitrogen, HDL-C=high-density lipoprotein cholesterol, TNF-α= tumor necrosis factor a, LDL-C=low-density lipoprotein cholesterol, UACR=creatinine ratio, Ucr=urinary creatinine, a means compared with the healthy group, P<0.05; b means compared with T2DM group, P<0.05.

Indicators	Healthy group <br>(n=50)	T2DM group <br>(n=50)	EDKD group <br>(n=50)	F	P
DBP (mmHg)	69.55±8.22	71.26±8.03	72.19±8.31	1.337	0.266
SBP (mmHg)	115.57±10.28	120.15±11.38	125.21±12.27	9.042	<0.001
TC (mmol/L)	4.58±0.66	4.60±0.69	4.66±0.55	0.214	0.808
TG (mmol/L)	1.65±0.53	1.71±0.49	1.95±0.63^ab^	4.118	0.018
LDL-C (mmol/L)	2.39±0.69	2.42±0.66	2.73±0.75^ab^	3.605	0.030
HDL-C (mmol/L)	1.55±0.43	1.33±0.42^a^	1.25±0.39^a^	7.051	0.001
hs-CRP (mg/L)	0.94±0.26	1.15±0.35^a^	1.92±0.57^ab^	77.540	<0.001
FPG (mmol/L)	6.01±0.54	9.22±1.11^a^	15.39±1.24^ab^	1114.000	<0.001
HbA1c (%)	5.61±0.66	8.29±0.73^a^	9.12±0.67^ab^	356.100	<0.001
FIns (mU/L)	7.75±2.01	13.21±2.05^a^	17.13±3.34^ab^	171.600	<0.001
HOMA-IR	3.48±1.02	6.15±2.02^a^	9.15±2.78^ab^	93.930	<0.001
UACR (mg/g)	9.03±4.67	11.16±5.01^a^	56.23±10.16^ab^	710.000	<0.001
BUN (mmol/L)	5.97±1.03	6.02±1.28	8.08±0.95^b^	60.370	<0.001
Ucr (μmol/L)	68.63±10.25	69.13±10.11	78.82±9.98^b^	16.130	<0.001
SOD (U/mL)	100.06±7.33	87.56±7.25^a^	73.15±8.26^ab^	155.900	<0.001
MCP-1 (μg/min)	76.33±7.21	93.26±9.13^a^	108.22±10.14^ab^	160.300	<0.001
TNF-α (pg/L)	31.25±4.79	35.23±5.86^a^	38.82±6.64^ab^	21.220	<0.001

### Comparison of three groups of NLRP3 inflammasome- related cells

The monocyte count of the three groups was *P*<0.001. The number of monocytes in the EDKD and T2DM groups was bigger than that in the healthy group (*P*<0.001 for all), and the number of monocytes in the EDKD group was bigger than that in the T2DM group, with *P*<0.001. See Table 2.

**Table 2:** Comparison of NLRP3 inflammatory vesicle-associated cells among the three groups (x̄±s). Note: ^d^ means compared with T2DM group, P<0.05. ^c^ means compared with the healthy group, *P*<0.05.

Time	White blood cell count (×10^9^/L)	Monocyte count (×10^9^/L)	Lymphocyte count (×10^9^/L)
Healthy group (n=50)	6.42±1.22	0.52±0.08	1.76±0.55
T2DM group (n=50)	6.53±1.11	0.64±0.04^c^	1.65±0.62
EDKD group (n=50)	6.77±0.79	0.72±0.06^cd^	1.54±0.67
F	1.437	131.000	1.598
*P*	0.241	<0.001	0.206

### Comparison of three groups of NLRP3 inflammasomes and related molecules

The levels of NLRP3, NT-proBNP and others in the three groups were compared, with P<0.001. Those in the EDKD and T2DM groups were bigger than the healthy group, with *P*<0.001 for all, and the levels of the above indicators in the EDKD group were higher than the T2DM group, with *P*<0.001 for all, as indicated in Table 3. This outcome data illustrated that NLRP3, NT-proBNP, caspase-1, ASC, IL-1β, and IL-18 levels were elevated in patients with T2DM and EDKD and that the above indicators were higher in patients with EDKD than in patients with T2DM.

**Table 3:** Comparison of three groups of NLRP3 inflammasomes and related molecules (x̄±s). Note: IL-1 β=Interleukin 1β IL-18=Interleukin 18. ^e^ means compared with the healthy group, *P*<0.05; ^f^ means compared with the T2DM group, *P*<0.05. NT-proBNP=amino B terminal type natriuretic peptide precursor, caspase-1=cysteine containing aspartic acid protein hydrolase 1, ASC=apoptosis related spot protein.

Indicator	The healthy group <br>(n=50)	T2DM group <br>(n=50)	EDKD group (n=50)	F	P	Indicator
NLRP3 (pg/mL)	318.25±77.30	611.51±68.55^e^	763.24±82.31^ef^	439.900	<0.001	NLRP3 (pg/mL)
NT-proBNP <br>(pg/mL)	34.25±18.14	964.35±55.76^e^	2451.21±1534.30^ef^	94.560	<0.001	NT-proBNP <br>(pg/mL)
caspase-1 <br>(pg/mL)	41.47±11.36	73.35±9.68^e^	116.28±10.49^ef^	635.200	<0.001	caspase-1 <br>(pg/mL)
ASC (pg/mL)	17.65±3.61	38.57±5.56^e^	62.19±8.85^ef^	609.200	<0.001	ASC (pg/mL)
IL-1β (pg/mL)	32.25±7.72	52.06±8.83^e^	83.15±10.37^ef^	402.900	<0.001	IL-1β (pg/mL)

### Correlation of NLRP3 inflammatory vesicle-associated cells and molecules with renal function indices in EDKD group

NLRP3, caspase-1, ASC, IL-1β, and IL-18 in the EDKD group were positively correlated with UACR (P:<0.001, <0.001, 0.001, <0.001, <0.001, <0.001), BUN (P: 0.012, 0.016, 0.009, 0.012, 0.024), and Ucr (P: 0.002, 0.003, 0.008, 0.023, 0.002) were positively correlated, and the differences were statistically significant (*P*<0.05) (see Table 4). This resultant data suggested that changes in the NLRP3 inflammatory vesicles and their related molecules were closely related to renal function in patients with EDKD.

**Table 4:** Correlation of NLRP3 inflammatory vesicles and their associated cells and molecules with other indicators. Note: * indicates *P*<0.05, TG=triglyceride, hs-CRP=high-sensitivity C-reactive protein, FPG=fasting blood sugar, HbA1c=glycated hemoglobin, SOD=superoxide dismutase, TNF-α= tumour necrosis factor α, UACR=creatinine ratio, BUN=blood urea nitrogen, IL-1β= Interleukin 1β, HDL-C=high-density lipoprotein cholesterol, MCP-1=monocyte chemotactic protein, LDL-C=low-density lipoprotein cholesterol, Ucr=urinary creatinine, TC=total cholesterol, ASC=apoptosis related spot protein, FIns=fasting insulin, NT-proBNP=amino B-terminal pro natriuretic peptide, caspase-1=cysteine containing aspartic acid protein hydrolase 1, HOMA-IR=insulin resistance, IL- 18=interleukin 18, SBP=systolic pressure, DBP=diastolic pressure.

Renal function <br>indicators	NLRP3	NT-proBNP	Caspase-1	ASC	IL-1β	IL-18	Mononuclear <br>cell count
UACR (mg/g)	0.600*	0.320	0.615*	0.470*	0.507*	0.581*	0.258*
BUN (mmol/L)	0.435*	0.286	0.512*	0.576*	0.554*	0.482*	0.218*
Ucr (μmol/L)	0.465*	0.412	0.550*	0.581*	0.503*	0.641*	0.086*

## Discussion

In this study, the baseline data of T2DM patients and EDKD patients were sorted out, and these indicators were compared with those of elderly people who passed the health physical examination in the same period. It was found that the T2DM group and EDKD group had abnormalities in SBP, TG, LDL-C, HDL-C, hs-CRP, FPG, HbA1c, FIns, HOMA-IR, UACR, BUN, Ucr, SOD, MCP-1, TNF-α and other indexes are abnormal, suggesting that T2DM patients and EDKD patients have different degrees of abnormalities of glucose, insulin, and lipids, as well as liver and kidney function damage. This abnormal change is more obvious in EDKD patients, which aligns with previous studies’ viewpoints [8]. Diabetic kidney disease is induced by abnormal glucose metabolism, and if treatment is delayed or poor, it will rapidly progress to the end stage of renal disease, and early diagnosis and early treatment are favourable to the prognosis [9]. It is important to explore the indicators related to the development and progression of diabetic kidney disease, including inflammation-related cells and molecules, to guide clinical preventive interventions and diagnosis.

Currently, the pathogenesis of diabetic kidney disease is still unclear, and most studies suggest that the development and progression of the disease are influenced by inflammation, metabolism, oxidative stress and other factors. It has been found that NLRP3 inflammatory vesicles are also involved [10]. NL-RP3 inflammatory vesicles have been shown to be an important factor in its ability to initiate the inflammatory response of the organism, which is normally in a suppressed state in the organism. Studies have shown that the inflammatory response is present in the onset and progression of diabetes, and disturbed glucose metabolism in diabetic patients promotes the synthesis of NLRP3 inflammatory vesicles, creating a chronic inflammatory response [11]. NLRP3 inflammatory vesicles originating from renal intrinsic cells are activated in glomerular endothelial cells and podocytes of diabetic patients [12]. Xu et al. [13] showed that FOXM1 activates deacetylase 4 during transcription and inhibits the nuclear factor B signalling pathway and NLRP3 inflammatory vesicles, attenuating renal injury and podocyte apoptosis in diabetic renal disease. He et al. [14] showed that NLRP3 inflammatory vesicle synthesis in diabetic patients is a key component of the inflammatory response. found that procyanidin B, a selective inhibitor of NLRP3 inflammatory vesicle activity, inhibits the assembly and activation of this inflammatory vesicle, which ultimately reduces urinary protein, blood creatinine, and blood urea nitrogen in mice with lupus nephritis (glomerulonephritis) model, as well as reduces immune complex deposition and glomerular injury in renal tissue. Chen et al. [15] found that a high glucose environment could promote ATP-P2X4 purinergic receptor axis-dependent extracellular ATP-mediated activation of NLRP3 inflammatory vesicles in renal tubular epithelial cells, catalyze the maturation and promote the secretion of IL-1β and IL-18, and thus participate in the inflammatory response of diabetic renal tubules. In EDKD patients, the synthesis and secretion of NLRP3 inflammatory vesicles NLRP3, ASC, and caspase-1 rise in their renal cells induced by high glucose, and the consumption of extracellular ATP by eATP hydrolase inhibits the up-regulation of NLRP3 inflammatory vesicles, which in turn attenuates the renal tubular inflammatory response. Another study [16] showed that in diabetic nephropathy, optic nerve proteins inhibit the activation of NLRP3 inflammatory vesicles by enhancing autophagy in renal tubular cells. It is suggested that there is an injurious effect of NLRP3 inflammatory vesicles on renal tubules. The inflammatory response dominated by NLRP3 inflammatory vesicles, in combination with the inflammatory factors IL-1β and IL-18, will promote the progression of chronic kidney disease in diabetic patients [17] [18]. Persistent glomerular and tubular injury in diabetic patients dramatically aggravates renal impairment to the point where the injury is irreversible, resulting in end-stage renal failure. In diabetic patients, NLRP3 expression is up-regulated, followed by activation of Caspase-1. Activated Caspase-1 continues to activate downstream proteins, prompting the active N-terminus of the downstream proteins to accumulate on the renal tubular cell membranes, forming cellular micropores, inducing apoptosis, and stimulating the synthesis and release of large quantities of inflammatory factors, such as IL-1β, IL-18 and other inflammatory factors, generating inflammatory cascade responses that inducing renal functional impairment [19]. Detecting the changes of NLRP3 inflammatory vesicles in EDKD has a certain significance in guiding the early diagnosis and improving the prognosis of EDKD in the elderly. In recent years, more and more studies have shown that the inflammatory response is an important factor in developing diabetic kidney disease [20] and that renal decompensation in diabetic patients is closely related to diabetes-induced renal interstitial inflammation [21]. IL-1β and IL-18 are representative members of the maintenance of the microinflammatory state of the body, which is closely related to the development and progression of diabetic kidney disease. The results of the present study confirmed this. In patients with T2DM, a hyperglycemic environment stimulates activated monocyte-macrophages to synthesize IL-1β and IL-18 in large quantities, which in turn activates renal tubular epithelial cell nuclear transcription factor (NF-KB), exacerbates inflammatory responses, and enhances the promotional effect of transforming growth factor-β on renal fibrosis [22].The precursor forms of IL-1β and IL-18 are inactive and need to be cleaved by caspase- 1 to exert a pro-inflammatory response, and caspase-1 is the end product of cleavage of pro-caspase- 1 by NLRP3. Monocytes, derived from hematopoietic stem cells in the bone marrow, are an important part of the body’s defence system. They participate in the immune response by phagocytosis of antigens and delivery of antigenic determinants. Inflammation in the body can cause changes in the total number and percentage of monocytes [23].

In the results of Pearson correlation analysis in this study, NLRP3, caspase-1, ASC, IL-1β, and IL-18 were significantly positively correlated with UACR, BUN, and Ucr in EDKD patients, respectively. It indicated that NLRP3 inflammatory vesicles and certain related cellular molecules were not only related to the occurrence of EDKD but also closely associated with the degree of progression of EDKD, which validated the previous view. However, the Pearson correlation analysis method used in this study could quantify the degree of correlation between variables through the correlation relationship and provide a more accurate metric for the study, which can accurately analyze the linear relationship between two variables. However, this research method can only describe the linear relationship between variables, not the causal one. In addition, this study is a single-center study with a small sample size, and the results may be biased, which needs to be further verified by expanding multicenter and increasing the sample size.

In summary, the levels of NLRP3 inflammasome and its related molecules, caspase-1, ASC, IL-1β, IL- 18, etc., were increased in early EDKD in the elderly and were significantly correlated with the severity of EDKD. NLRP3 inflammasome and its related molecules may be involved in renal acute and chronic inflammatory responses, contributing to the formation of early EDKD. An in-depth study of their activation and regulation mechanisms may provide new ideas for the early differential diagnosis and treatment of EDKD.

## Dodatak

### Funding

The research is supported by the Shanxi Basic Research Program – GLP-1 promotes podocyte mitochondrial autophagy to improve early diabetic nephropathy and its mechanism (NO: 202203021212102).

### Conflict of interest statement

All the authors declare that they have no conflict of interest in this work.
